# Generative AI Engagement and Perceived Nutrition-Oriented Feeding Practices Among Urban Indonesian Mothers: Mixed Methods Study

**DOI:** 10.2196/100061

**Published:** 2026-09-21

**Authors:** Lia Nur Amalina, Ferdi Antonio, Surya Adiwena, Dewi Wuisan, Roy Glenn Massie

**Affiliations:** 1 Department of Hospital Administration, Graduate School of Management, Pelita Harapan University, Jl. Garnisun Dalam No. 8, RT 5, RW 4, Karet Semanggi, Jakarta, Jakarta Special Capital Region, 12930, Indonesia, 62 812-1010-676; 2 Health Policy, National Research and Innovation Agency (BRIN), Jakarta, Indonesia

**Keywords:** generative artificial intelligence, mothers, feeding behavior, parenting, parental self-compassion, cognitive empowerment, self-efficacy, health literacy, Indonesia

## Abstract

**Background:**

The Asia-Pacific region faces an escalating double burden of malnutrition, with childhood obesity now affecting more than 113 million children aged 0‐19 years. A demographic of highly educated, digitally literate urban mothers—referred to in this study using the descriptive label “Alpha Mother”—increasingly draws on generative AI as a source of toddler nutrition information. Yet, whether this shift empowers or undermines quality feeding practices remains empirically untested.

**Objective:**

This study examined how generative artificial intelligence (AI) engagement is associated with perceived nutrition-oriented feeding practices among urban Alpha Mothers in Indonesia, with cognitive empowerment and parental self-compassion as intervening variables, and eHealth self-efficacy as a moderator. The study contextually refined 5 AI engagement dimensions, organized through an AI parenting nutrition alignment approach.

**Methods:**

An exploratory sequential mixed methods design was used. Phase 1 comprised semistructured interviews with 10 Alpha Mothers and used hybrid deductive-inductive thematic analysis to contextually refine 5 literature-informed AI engagement constructs: AI algorithmic trust, AI health information quality, AI personalization fit perception, AI information-seeking intensity, and AI social proof sensitivity. These informed a survey instrument developed through the qualitative phase and refined against previously validated scales in phase 2, recruiting 442 respondents across 4 Indonesian urban centers. A 14-hypothesis structural model was tested using PLS-SEM (partial least squares–structural equation modeling)

**Results:**

Twelve of 14 hypotheses were supported. The model demonstrated substantial explanatory power for cognitive empowerment (*R*²=0.661) and parental self-compassion (*R*²=0.719), while perceived nutrition-oriented feeding practices yielded a more modest value (*R*^2^=0.280). This result reflects the multifactorial nature of self-reported nutrition-oriented feeding practices, which is shaped by numerous personal, cultural, and situational factors beyond AI engagement. AI algorithmic trust showed the strongest association with parental self-compassion (*β*=.479; *P*<.001), while AI personalization fit perception most strongly predicted cognitive empowerment (*β*=.275; *P*<.001). Both intervening variables were significantly associated with nutrition-oriented feeding practices, with cognitive empowerment (*β*=.232; *P*=.003) outperforming parental self-compassion (*β*=.165; *P*=.009). eHealth self-efficacy moderated the cognitive empowerment pathway (*β*=.124; *P*=.02) but not the self-compassion pathway.

**Conclusions:**

The findings suggest that generative AI engagement is associated with perceived nutrition-oriented feeding practices via 2 distinct indirect associations examined in this study: cognitive empowerment and parental self-compassion. These associations were strongest when mothers trusted the AI system and perceived its recommendations as fitting their child and household realities, indicating that the perceived value of AI in toddler feeding may lie less in algorithmic sophistication than in its capacity to build trust, fit context, and support maternal judgment. Because the outcome reflects maternal perception rather than observed feeding behavior or child nutrition outcomes, these findings should be interpreted as associational. They may nonetheless inform future research on AI-assisted feeding support that complements, rather than replaces, professional nutrition counseling.

## Introduction

Southeast Asia continues to face a double burden of malnutrition, with persistent undernutrition coexisting alongside rapidly increasing childhood overweight and obesity [1]. In East Asia and the Pacific, more than 113 million children aged 0‐19 years are overweight or obese, with Indonesia among the countries substantially affected [2,3]. Early dietary patterns established during the toddler period play an important role in shaping lifelong eating behaviors and health outcomes, making caregiver feeding practices an important target for public health intervention [4]. The quality of these early feeding practices, rather than obesity as a measured outcome, is the focus of this study. Accordingly, the World Health Organization (WHO) and the United Nations Children’s Fund (UNICEF) identify responsive feeding as a cornerstone of optimal infant and young child nutrition, emphasizing reciprocal caregiver-child interactions that encourage autonomous eating, healthy food preferences, and appropriate responses to hunger and satiety cues [5]. Consistent with this principle, the WHO Guideline for Complementary Feeding of Infants and Young Children aged 6‐23 months recommends responsive feeding alongside continued breastfeeding, age-appropriate complementary feeding, dietary diversity, and limiting unhealthy foods and beverages [6]. These recommendations provide an important benchmark for evaluating how emerging digital technologies may support or potentially undermine maternal feeding decisions.

Despite these recommendations, maintaining responsive feeding has become increasingly challenging for many Indonesian mothers who simultaneously balance employment, caregiving responsibilities, and growing exposure to digital nutrition information and commercial food marketing [7-10]. Social media further amplifies conflicting dietary advice, increasing cognitive demands and making it more difficult to distinguish evidence-based recommendations from misinformation [11,12]. Contemporary motherhood literature describes an emerging demographic of highly educated urban mothers who combine professional identities with intensive parenting practices centered on optimizing children’s development [13,14]. To characterize this population, this study adopts the descriptive label “Alpha Mother,” a term previously used in motherhood literature to describe technologically engaged mothers who actively seek expert guidance for child-rearing decisions [15]. Related scholarship further suggests that online parenting environments reinforce intensive mothering ideals through social comparison and curated self-presentation [16,17]. In this study, the term refers specifically to urban Indonesian mothers with tertiary education, relative economic stability, and active use of generative artificial intelligence (AI) for toddler nutrition information. It is used solely as an analytical descriptor of the study population rather than as a normative ideal of motherhood.

As digital health technologies become increasingly integrated into parenting, Alpha Mothers are also adopting generative AI platforms as convenient sources of personalized nutrition information [18-24]. Unlike conventional search engines, conversational AI can provide interactive, context-sensitive responses that may help mothers navigate complex feeding decisions while managing substantial cognitive demands. However, these potential benefits remain accompanied by important limitations. AI-generated health information is highly dependent on user prompting and remains susceptible to hallucinations, inaccurate recommendations, and embedded biases [25-27]. Furthermore, although AI may complement evidence-based resources such as the WHO and UNICEF Community Infant and Young Child Feeding (C-IYCF) Counselling Package, its role in supporting maternal decision-making has not yet been sufficiently established.

Beyond information quality, the conversational nature of generative AI introduces additional psychological considerations. Human-like responses may encourage users to attribute credibility, empathy, and emotional understanding to AI systems despite their lack of genuine interpersonal awareness—a phenomenon referred to as pseudoempathy [28,29]. Such interactions may foster algorithmic authority, whereby users increasingly defer health-related decisions to algorithmic recommendations rather than their own judgment or professional advice [30-34]. While traditional maternal feeding research has largely relied on knowledge-attitude-practice frameworks [35], these models do not fully capture AI-mediated parenting decisions in which trust, cognitive burden, and perceived personalization influence how caregivers interpret health information [27,36-39]. Consequently, understanding maternal engagement with generative AI requires conceptual approaches that extend beyond conventional behavioral models.

Although digital motherhood, AI-assisted health communication, and parenting technologies have received increasing scholarly attention, little empirical research has examined how maternal engagement with generative AI relates specifically to perceived nutrition-oriented feeding practices within real-world parenting contexts. To address this gap, this study proposes the AI Parenting Nutrition Alignment Approach, a conceptual framework informed by prior literature and contextually refined through the qualitative phase of this mixed methods study. Drawing on Sánchez-Vera’s [40] 3 dimensions of algorithmic agency—structural, operational, and symbolic—the approach describes how AI-generated nutrition advice aligns with mothers’ child characteristics, cultural context, household circumstances, and parenting values. Rather than introducing entirely new behavioral theories, this framework organizes the 5 AI engagement dimensions refined through a hybrid deductive-inductive thematic analysis conducted during phase 1 into a coherent structure for subsequent quantitative evaluation. The study’s primary contribution therefore lies not in proposing new theoretical constructs but in operationalizing these dimensions and demonstrating their convergence with established constructs within this specific parenting context.

**Figure 1.**
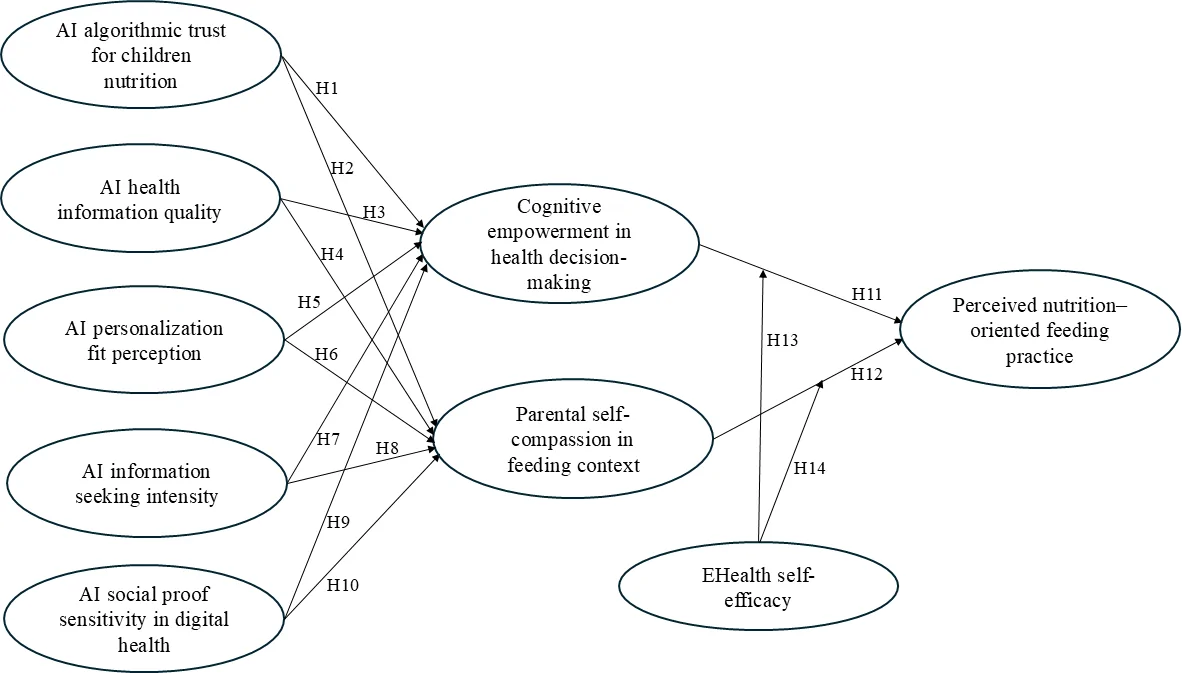
Conceptual framework. AI: artificial intelligence.

Building upon the AI Parenting Nutrition Alignment Approach, this exploratory sequential mixed methods study investigates how generative AI engagement is associated with perceived nutrition-oriented feeding practices among urban Indonesian Alpha Mothers. Specifically, the study examines whether these relationships are indirectly associated via the complementary constructs of cognitive empowerment and parental self-compassion, while evaluating eHealth self-efficacy as a potential moderator. Guided by these objectives, 2 research questions were formulated. Research question 1: To what extent is generative AI engagement associated with the 2 intervening constructs—cognitive empowerment and parental self-compassion—and, in turn, with perceived nutrition-oriented feeding practices? Research question 2: Does eHealth self-efficacy serve as a boundary condition in the indirect associations involving these constructs to perceived nutrition-oriented feeding practices? To address these questions, 14 hypotheses were developed. The 5 AI engagement dimensions refined through the qualitative phase—AI algorithmic trust, AI health information quality, AI personalization fit perception, AI information-seeking intensity, and AI social proof sensitivity—were hypothesized to be positively associated with cognitive empowerment and parental self-compassion (hypothesis [H]1 to H10). Both intervening constructs were further hypothesized to be associated with perceived nutrition-oriented feeding practices (H11 to H12), while eHealth self-efficacy was hypothesized to moderate both construct–outcome relationships (H13 to H14). The resulting conceptual framework is presented in Figure 1.

## Methods

### Study Design

This study used a mixed methods exploratory sequential design [41]. The initial qualitative phase explored Alpha Mothers’ interactions with AI to understand maternal emotional dynamics and to refine and contextualize key constructs. These findings directly informed the subsequent quantitative phase by guiding item development, refining definitions, and contextualizing scale wording to reflect actual feeding experiences. Finally, the second phase empirically tested the derived structural model and research hypotheses within a larger population sample. The resulting conceptual framework, which maps the 14 proposed hypotheses and structural pathways, is illustrated in Figure 1.

### Mixed Methods Integration: From Qualitative Themes to Quantitative Constructs

Consistent with the exploratory sequential design, the 2 phases were connected at an explicit point of interface: the 5 literature-informed domains were contextually refined in phase 1, and the resulting subthemes and indicators informed the constructs measured in phase 2. Each of the 5 AI engagement dimensions—algorithmic trust, health information quality, personalization fit perception, information-seeking intensity, and social proof sensitivity—was operationalized into a measurable construct with a corresponding conceptual definition and a dedicated set of survey items.

The quantitative instrument was developed in several stages. Items were developed from the context-specific subthemes and indicators identified in phase 1 and refined with reference to previously validated instruments measuring related constructs. Before data collection, all items underwent face validity review by 2 subject-matter experts to assess clarity and relevance. The instrument was then pilot-tested with 30 respondents meeting the study eligibility criteria to evaluate item readability and preliminary internal consistency (Cronbach α). Based on the pilot results, several items were reworded for clarity and 1 item was removed before finalizing the instrument. These 30 pilot respondents were not included in the main analytic sample (N=442). All measurement items used a 5-point Likert response format.

### Recruitment and Participant Demographics

The target population, referred to using the descriptive label “Alpha Mothers” as defined in the Introduction section, comprised urban mothers actively engaging with generative AI for child-related information. Eligibility was determined through a 2-stage process: a brief prescreening confirmed current parenting of a child aged 6‐59 months and prior AI use, while remaining criteria (duration and frequency of AI use, urban residence, and exclusion of health care professionals) were verified through the main survey and manual confirmation by the first author during recruitment. The complete screening and verification items are presented in Multimedia Appendix 1. The geographic scope covered Greater Jakarta, Bandung, Surabaya, and Medan.

For the qualitative phase, recruitment was conducted using purposive and snowball sampling techniques across diverse urban domiciles. The targeted sample size was established at 10-20 key informants, with interviews continuing until theoretical data saturation was achieved and no new themes emerged. In the quantitative phase, participants were recruited via a nonprobability purposive sampling approach.

Outreach efforts strategically used digital parenting communities, pediatric clinic networks, and school communities, channels deliberately selected for their alignment with the digitally literate profile of the target population with implications of this approach for sample composition discussed in the Limitations section. Sample size was determined a priori using G*Power (version 3.1.9.7; Heinrich Heine University Düsseldorf) [42]. Following a recommendation by Hair et al [43], the minimum sample size for PLS-SEM (partial least squares–structural equation modeling) was estimated based on the maximum number of predictors converging on a single endogenous construct in the structural model. The most complex regression equation in the model corresponded to the cognitive empowerment and parental self-compassion constructs, each receiving 5 direct paths from exogenous variables (AI Algorithmic Trust, AI Health Information Quality, AI Personalization Fit, AI Information-Seeking Intensity, and AI Social Proof Sensitivity). With *f*²=0.15 (medium effect size), α=.05, and statistical power=0.90, the minimum required sample size was 116 respondents. To ensure proportional geographic representation across the designated urban centers, stratified purposive sampling with regional quotas was applied.

Inclusion criteria stipulated that participants must (1) be mothers residing in the designated urban areas; (2) have at least 1 child aged 6-59 months; (3) actively use generative AI platforms (eg, ChatGPT, Gemini, Grok, etc) for at least 6 months, with a minimum frequency of 3 times per week, to seek childcare and nutritional information; and (4) provide voluntary informed consent to participate in the study. Mothers employed as health care professionals (eg, physicians, nurses, or nutritionists) were excluded from the study. This was a deliberate purposive sampling decision rather than a convenience-based one. Because the study examines how nonexpert mothers perceive, trust, and appraise AI-generated nutrition information, the inclusion of clinically trained mothers would have introduced a systematic confound: such participants evaluate health information through specialized professional knowledge and established clinical standards that differ fundamentally from the lay appraisal processes this study sought to capture [44,45]. Excluding them preserved the conceptual homogeneity of the target population and ensured that the measured constructs reflected lay maternal perception rather than professional clinical judgment. This exclusion was verified manually by the first author (LNA) during recruitment through direct confirmation of occupational status with each potential participant, in addition to the occupation question captured in the main survey. The complete eligibility screening and verification procedure is presented in Multimedia Appendix 1.

### Data Collection Procedures

Data collection was conducted sequentially across 2 distinct phases. During the qualitative phase, the first author (LNA) conducted all semistructured in-depth interviews face-to-face over a 3-week period in December 2025. All study materials were administered in Bahasa Indonesia. Interview quotations were translated into English using AI-based tools and verified for meaning equivalence by 2 bilingual team members (FA and SA) against the original transcripts. AI tools were used only for translation and language editing; all coding and thematic analyses were conducted manually. As the quantitative instrument was administered in Bahasa Indonesia, the English wording of the measurement items is for reporting only and did not affect measurement validity. A semistructured guide comprising 16 open-ended questions across 5 core domains (AI trust formation, information quality evaluation, personalization expectations, information-seeking behaviors, and social validation) directed each session, with flexible probing questions to elicit deeper reflection. With participant consent, all sessions were audio-recorded and transcribed verbatim, each lasting 30-45 minutes. To enhance credibility, synthesized member checking following the approach by Birt et al [46] was conducted, returning summarized thematic findings to participants for confirmation.

**Figure 2.**
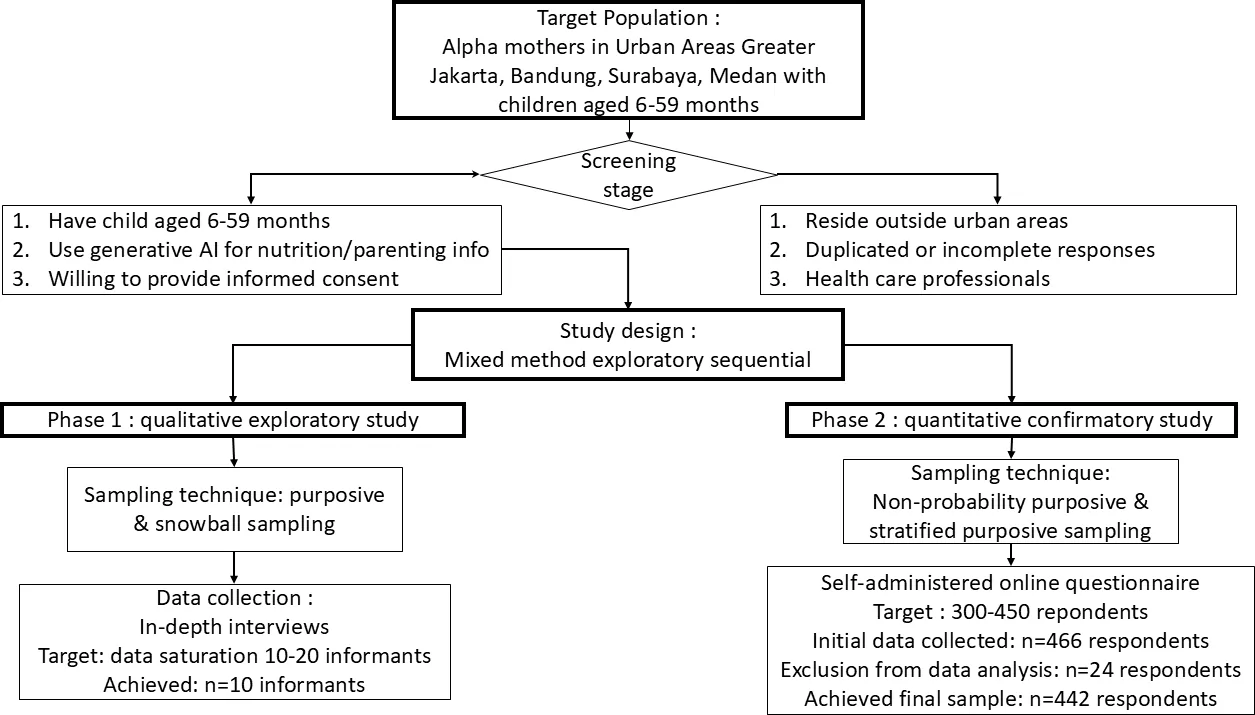
Sampling framework flowchart. AI: artificial intelligence.

The quantitative phase data were collected using a self-administered online questionnaire. This survey commenced with a brief screening section to verify that respondents met the designated “Alpha Mother” criteria. Several procedural remedies were implemented to mitigate common method bias (CMB), including randomization of item order within construct blocks, use of psychological separation between predictor and criterion measures, and ensuring respondent anonymity to reduce social desirability concerns [47,48]. Finally, rigorous data-cleaning protocols, including IP address tracking to identify and remove duplicate entries, were implemented prior to statistical analysis. An overview of the data collection process can be seen in Figure 2.

### Statistical Analysis

Qualitative data from the phase 1 interviews were analyzed using Braun and Clarke’s [49] 6-phase thematic analysis framework [50]. Because the interview guide was informed by 5 predefined conceptual domains from prior literature, the analysis followed a hybrid deductive-inductive approach. The predefined domains provided an initial structure for organizing the data, while open coding was used to identify context-specific meanings, indicators, and subthemes within and across those domains. Initial coding was conducted manually by the third author (SA), generating 29 initial codes that were collaboratively grouped into 13 subthemes and consolidated into 5 overarching themes. The coding framework and thematic structure were subsequently reviewed and refined through consensus discussion with LNA and FA to ensure conceptual coherence and that the themes remained grounded in participants’ accounts. A complete audit trail linking the predefined domains, interview questions, initial codes, subthemes, final themes, construct definitions, and survey items is provided in Multimedia Appendix 2. Data saturation was assessed iteratively rather than assumed. After each interview, the research team evaluated whether new codes, themes, or subthemes were emerging. No substantive new themes or subthemes were identified across the final 3 consecutive interviews (interviews 8‐10), indicating that thematic saturation had been reached by the 10th interview. This sample size is consistent with empirical guidance indicating that thematic saturation is typically achieved within 9-17 interviews in studies involving relatively homogeneous populations and narrowly defined research objectives [51], conditions that characterize this study’s focus on urban Alpha Mothers.

The first author (LNA), who conducted all interviews, is a mother with personal experience using AI for parenting decisions. This shared identity not only facilitated rapport and nuanced understanding of participants’ experiences but also introduced potential for overidentification. To mitigate this risk, initial coding was conducted by the third author (SA), who was not involved in conducting the interviews. The coding framework, subthemes, and final themes were subsequently reviewed and refined through consensus discussions with LNA and FA, helping ensure that interpretations remained grounded in participants’ accounts.

Quantitative data from phase 2 were analyzed using PLS-SEM via SmartPLS 4 software (SmartPLS GmbH), following the evaluation guidelines by Hair et al [43]. The evaluation was conducted in 2 primary stages: the measurement model (outer model) and the structural model (inner model). For the measurement model, indicator reliability was assessed using outer loadings. Construct reliability was established using both Cronbach α and composite reliability (CR), while convergent validity was evaluated through the average variance extracted. Discriminant validity was assessed using the Heterotrait-Monotrait (HTMT) ratio of correlations, with thresholds and interpretation criteria following the guidelines of Hair et al [43,52].

Following a satisfactory measurement model, the structural model was evaluated. The overall quality and predictive relevance of the inner model were assessed by examining the inner variance inflation factor (VIF) for collinearity issues, the coefficient of determination (*R*²), Q²_predict_ values, and Cross-Validated Predictive Ability Test (CVPAT) [53,54]. Subsequently, a bootstrapping procedure was applied to test the significance of the 14 proposed research hypotheses, including the moderation effects. Following the a priori structural model, the 5 AI engagement constructs were linked to perceived nutrition-oriented feeding practices only via cognitive empowerment and parental self-compassion; direct AI engagement-to-outcome paths were not estimated. Consequently, the present analysis does not distinguish direct from indirect associations, and results are interpreted solely as indirect associations rather than as evidence of mediation. Indirect effects were tested using bias-corrected bootstrap confidence intervals, and an effect was considered supported when its 95% CI excluded zero. Finally, an Importance-Performance Map Analysis (IPMA) was conducted to generate actionable insights regarding priority areas for digital health interventions. To assess whether the modeled relationships were consistent across child developmental stages, a bootstrap-based multigroup analysis (MGA) was additionally conducted, comparing mothers of children aged 6‐24 months with those of children aged 25‐59 months (Multimedia Appendix 3) [43,55].

### Ethical Considerations

All research procedures were conducted in accordance with strict ethical principles for medical and social research. The study protocol was reviewed and approved by the Research Ethics Committee (KEP FEB), Faculty of Economics and Business, Universitas Pelita Harapan, under approval number 047/MARS/EC/XI/2025, issued on November 26, 2025. All recruitment and data collection commenced after this approval was granted. Prior to participation, all individuals were provided with a comprehensive participant information sheet detailing the study’s objectives, guarantees of data anonymity and confidentiality, and their right to withdraw at any time without penalty. Explicit informed consent obtained either in writing for the interviews or electronically for the survey was mandatory before any data collection occurred.

## Results

### Phase 1: Qualitative Exploratory Study

#### Participant Characteristics

In the first phase of this qualitative study, 10 informants were selected through in-depth interviews until data saturation was reached. All informants were Alpha Mothers with children aged 6‐59 months, residing in Indonesia’s urban centers, including Greater Jakarta, Bandung, Surabaya, and Medan, and frequently using AI as their health care assistant. The informants’ characteristics indicated a middle-to-upper socioeconomic profile with a high level of education.

All 10 participants subsequently engaged in the synthesized member-checking process and confirmed that the thematic interpretations accurately reflected their experiences as urban Indonesian mothers navigating AI-assisted child nutrition decisions. No substantive disagreements emerged, and the thematic structure was carried forward to operationalization for the phase 2 quantitative instrument. The participants’ employment dynamics were quite diverse, consisting of housewives (n=3), full-time workers (n=3), freelancers (n=2), and professionals (n=2). Most informants had 2 children (n=5), while the remainder had 1 child (n=5), providing a broad perspective from both new and more experienced mothers. Regarding digital literacy, all informants had extensive experience using generative AI (all had more than 6 months of AI use). ChatGPT was the primary platform universally used by all informants, with some participants using additional platforms such as Gemini (n=4), Claude (n=1), and Grok (n=1) to validate children’s nutritional information.

#### Thematic Findings

Hybrid deductive-inductive thematic analysis organized the findings across 5 predefined domains and identified 13 context-specific subthemes (Table 1). These themes explore how Alpha Mothers evaluate, validate, and integrate AI-generated information into their toddler-feeding routines, directly establishing the foundation for the quantitative structural model in phase 2. The first major theme, AI Algorithmic Trust, highlights that maternal trust in AI does not emerge automatically; rather, it is contingent upon perceived technical competence (T1) and moral integrity (T2). Mothers position AI not merely as a search engine but as a stable “thinking partner” expected to provide objective advice free from commercial bias. As one mother emphasized, *“*I need honest advice...solely focused on my child’s health, no commercial bias” (I1). Furthermore, mothers expect the algorithm to align with their core parenting values (T3), seeking an AI that “has the same values and goals” (I8) while maintaining a “strong scientific basis” (I3).

**Table 1.** Thematic exploratory.

Theme	Respondent quotes	Conceptual interpretation
AI^a^ Algorithmic Trust		
T1: Competency and Reliability	“I need a thinking partner who is stable, unchanging, and this AI is competent...has a strong scientific basis.” [I3]	The perception that AI is a competent and stable expert provides a sense of security, such as technical trust for users.
T2: Moral Integrity and Objectivity	“I need honest advice...solely focused on my child’s health, no commercial bias.” [I1]	The perception that AI provides honest, genuine advice free from commercial advertising bias.
T3: Value Alignment	“Must have the same values and goals as me...just like chatting with friends on the same wavelength.” [I8]	The degree of conformity between AI logic and the parenting principles held by the mother.
AI Health Information Quality		
T4: Accuracy and Completeness	“The data must be accurate...really look for accuracy. It must be complete and thorough...from measurements to allergy risks.” [I8]	Information must be proven to be medically accurate and presented in a complete manner.
T5: Relevance	“It’s useless if it’s not relevant to my child’s current condition.” [I3]	The accuracy of information in answering specific questions with the user’s current conditions.
T6: Understandability	“Don’t use complicated language...medical jargons or (seemingly) otherworldly language.” [I2]	Use simple language and do not contain complicated medical language.
AI Personalization Fit		
T7: Biological Fit	“I really don’t like it when I am given a menu that doesn’t suit my little brother’s situation... he has allergies...I was even given risky ingredients.” [I2]	AI’s ability to adapt suggestions to a child’s physical limitations for child safety.
T8: Culture and Taste Fit	“Our palate is local...if the food tastes too foreign or too western, children will definitely be turned off.” [I2]	Align menu suggestions with local tastes and family eating habits
T9: Practical Fit (Time and Budget)	“It fits the kitchen budget...It fits our time and daily eating habits.” [I7]	The level of realism of suggestions regarding the availability of time, energy, and mother’s shopping budget.
AI Information-Seeking Intensity		
T10: Search Frequency and Immediate Response	“Going back and forth opening the application, asking again, and again...I have to act quickly, I need an emergency solution right away.” [I10]	A behavioral pattern of asking questions repeatedly in a short period of time with the demand for an immediate response.
T11: In-Deep Exploration and Verification Effort	“Keep checking and comparing the answers to be sure. Dig deeper, not just about the menu...but also psychological tricks.” [I5]	Efforts to dig deep into the root of the problem while still actively verifying the accuracy of the data.
AI Social Proof Sensitivity		
T12: Mass Acceptance and Herd Safety	“Knowing that this AI is already widely accepted...thousands of other mothers are already using it. I don’t want my child to be a guinea pig...there’s a sense of security in being in the same group.” [I4]	The sense of security that arises from knowing that this technology is already used by the majority of people or mass acceptance.
T13: Validation And Consensus Reliance	“Very necessary for social validation...honest reviews, testimonials...need confirmation from the majority of other users confirming the safety.” [I3]	The need for confirmation from a third party in the form of personal testimony or expert opinion before finally being able to trust AI input.

a AI: artificial intelligence.

This reliability must be substantiated through rigorous AI Health Information Quality. Mothers demand comprehensive clinical accuracy (T4) and direct relevance to their specific situation (T5), coupled with high understandability (T6). They require thorough data, “from measurements to allergy risks” (I8), while strictly noting that any advice is “useless if it’s not relevant to my child’s current condition” (I3). Furthermore, to ensure that the information is easily comprehensible, the AI must avoid “medical jargon or (seemingly) otherworldly language” (I2).

Furthermore, objective medical quality must be complemented by AI personalization fit. AI recommendations must be adaptable to the user’s specific biological (T7), cultural (T8), and practical realities (T9). Biologically, mothers express strong dissatisfaction when AI fails to account for a child’s physical limitations, such as suggesting “risky ingredients” for a child with allergies (I2). Culturally, suggestions must align with local palates to avoid children being “turned off” by overly Westernized foods (I2). Finally, on a practical level, these recommendations must strictly conform to the mother’s “kitchen budget,” available time, and established daily eating habits (I7).

Interestingly, despite their high digital literacy, Alpha Mothers exhibit pronounced AI Social Proof Sensitivity. There is a distinct reluctance to adopt algorithmic advice without mass acceptance or third-party validation (T12, T13). The psychological safety of adopting AI relies heavily on herd consensus, driven by the fear of making their child a “guinea pig,” thus requiring “honest reviews, testimonials...need confirmation from the majority of other users confirming the safety” (I3, I4).

Finally, this comprehensive evaluation of trust, quality, and social validation drives AI Information Seeking Intensity (T10, T11). During critical situations, such as sudden toddler-feeding strikes, mothers demand immediate, “emergency solutions” (I10). However, they do not accept these answers passively; instead, they engage in intense, repetitive probing to “dig deeper, not just about the menu...but also psychological tricks,” while continuously cross-checking the data for accuracy (I5).

While participants valued AI as a feeding-support tool, their accounts also surfaced consistent negative and critical experiences. The most consequential concerned safety: several mothers reported that AI recommended ingredients unsuitable for their child’s specific medical condition. One mother described that for her allergic child, the AI “even offered risky ingredients” (I2), and another recounted that it suggested a menu whose ingredients “could actually risk making my child sick” (I10). Beyond safety, mothers expressed frustration when AI acted as if it “knew best” yet remained insensitive to their real situation at home (I1, I8), produced culturally mismatched menus that children refused so that the food “ended up wasted” (I3), or kept slipping in hard-to-find ingredients even after being explicitly prompted to avoid them (I6). These experiences indicate that mothers did not engage with AI uncritically; rather, they actively detected, questioned, and worked around its limitations.

These qualitative findings directly informed the quantitative phase of the study. The contextually refined themes and subthemes regarding mothers’ interactions with AI were systematically operationalized into measurable constructs. Table 2 outlines the conceptual definitions of these constructs, which served as the foundation for the survey instrument and structural model testing in phase 2.

**Table 2.** Construct definition.

Constructs	Conceptualized definition
AI^a^ Algorithmic Trust for Children Nutrition	This construct represents the mother’s psychological belief in AI technology for child nutrition which, within the AI parenting nutrition alignment approach, emerges from both a rational assessment of the system’s technical reliability and an emotional conviction regarding its integrity and its capacity to provide reassurance consistent with her parenting values [56].
AI Health Information Quality	This construct captures the mother’s evaluation of AI-generated nutritional information, focusing specifically on how she perceives its accuracy, completeness, and understandability [21,27].
AI Personalization Fit Perception	AI Personalization Fit Perception reflects the mother’s evaluation of how effectively the AI tailors its nutritional guidance to her specific reality. This encompasses her child’s biological health status, her own psychological preferences, and her immediate environmental or situational context [57].
AI Information-Seeking Intensity	AI Information-Seeking Intensity represents the frequency, duration, and depth of mother’s engagement with AI to obtain and verify nutritional information [58,59]. Within this study, a high intensity of use reflects the mother’s active alignment efforts to navigate and resolve digital uncertainty.
AI Social Proof Sensitivity	AI Social Proof Sensitivity characterizes a mother’s responsiveness to collective validation signals, such as app reviews, ratings, and overall popularity [60,61]. Within the AI parenting nutrition alignment approach, this reflects a herd—safety tendency, illustrating how mothers seek safety in numbers as a form of collective validation before adopting new health technologies.
Cognitive Empowerment in Health Decision-Making	Cognitive Empowerment in Health Decision-Making refers to a mother’s psychological and cognitive capacity to understand, evaluate, and use AI-generated information for practical toddler nutrition decisions. This construct is heavily dependent on her language comprehension and critical appraisal skills [36,62].
Parental Self-Compassion in Feeding Context	Adapted from Hasyyati and Abidin [63], this construct captures a mother’s ability to approach toddler-feeding challenges with self-compassion, which operationally involves practicing self-kindness, viewing feeding setbacks through the lens of common humanity, and sustaining mindfulness to preserve emotional balance.
eHealth Self-Efficacy	eHealth Self-Efficacy refers to a mother’s perceived confidence in her ability to effectively find, evaluate, and resolve toddler-feeding challenges using AI and other digital technologies [22,26].
Perceived Nutrition-Oriented Feeding Practices	Perceived Nutrition-Oriented Feeding Practices reflects a mother’s subjective evaluation of the nutrition-focused feeding strategies she implements for her child, distinct from observed feeding behavior or objective child nutrition outcomes. Operationally, this encompasses meal schedule consistency, deliberate nutritional appraisal of foods offered, limitation of low-nutrient foods, and attentiveness to the child’s satiety signals. Within the food parenting practices content map, these correspond primarily to the structure domain and, in part, to nutrition education within autonomy support; the construct does not operationalize coercive control practices or child autonomy support in full and therefore does not represent the complete responsive feeding construct as defined by WHO^b^ and UNICEF^c^ [64-66].

a AI: artificial intelligence.

b WHO: World Health Organization.

c UNICEF: United Nations Children’s Fund.

### Phase 2: Quantitative Explanatory Predictive Study

The second phase of this study used a quantitative survey to empirically validate the structural model derived from the qualitative findings. Specifically, this phase evaluated perceived nutrition-oriented feeding practices, operationalized as maternal self-report, not observed feeding behavior or objective child nutrition outcomes. The model then tested how nutrition-oriented feeding practices are associated with 5 AI-related predictors via 2 intervening constructs, cognitive empowerment (CE), and parental self-compassion (PSC), with eHealth self-efficacy (ESE) as a moderator.

#### Participant Characteristics

A total of 442 respondents completed the survey and met all inclusion criteria, substantially exceeding the minimum sample size of 116 derived from the a priori power analysis. This sample size provided robust statistical power for testing the proposed structural model. Geographic distribution was concentrated in the Greater Jakarta area (255/442, 57.7%). Demographically, the largest segment of mothers was aged 31-35 years (184/442, 41.6%).

Reflecting the “Alpha Mother” profile, over half held a bachelor’s degree (227/442, 51.4%), with a substantial proportion possessing postgraduate degrees (155/442, 35.1%). Socioeconomically, the predominant employment status was full-time office worker (139/442, 31.4%), followed closely by professionals (111/442, 25.1%). Routine monthly household expenditure primarily fell between US $292.18 and US $584.36 (217/442, 49.1%). Regarding family dynamics, nearly half of the participants had 2-3 children (217/442, 49.1%), and the target toddlers were most commonly aged between 37 and 59 months (169/442, 38.2%). ChatGPT emerged as the most frequently used AI platform for health information (259/442, 58.6%), followed by Gemini (125/442, 28.3%). A comprehensive breakdown of these participant demographics and characteristics is presented in Table 3.

**Table 3.** Participant demographics and characteristics (N=442).

Characteristics	Participants
City of residence, n (%)	
Greater Jakarta	255 (57.7)
Surabaya	64 (14.5)
Bandung	63 (14.3)
Medan	60 (13.6)
Mother’s age (years), n (%)	
18‐25	15 (3.4)
26‐30	148 (33.5)
31‐35	184 (41.6)
36‐40	95 (21.5)
Education level, n (%)	
Diploma (D3/D4)	60 (13.6)
Bachelor’s degree (S1)	227 (51.4)
Postgraduate degree (S2/S3)	155 (35.1)
Employment status, n (%)	
Full-time worker (office/WFO)	139 (31.4)
Professional	111 (25.1)
Housewife	99 (22.4)
Entrepreneur	53 (12)
Part-time/freelance	40 (9)
Monthly household expenditure, n (%)	
$292.18 to $584.36	217 (49.1)
$584.36 to $1168.73	92 (20,8)
>$1168.73	133 (30.1)
Number of children, n (%)	
1	202 (45.7)
2‐3	217 (49.1)
>3	23 (5.2)
Target child’s age (months), n (%)	
6‐24	136 (30.8)
25‐36	137 (31)
37‐59	169 (38.2)
Most frequently used AI^a^ platform, n (%)	
ChatGPT	259 (58.6)
Gemini	125 (28.3)
Claude	25 (5.7)
Grok	14 (3.2)
Copilot	10 (2.3)
Bing	9 (2)

a AI: artificial intelligence.

#### AI Usage for Child Nutrition

To understand exactly how these mothers relied on AI for toddler feeding, we asked them to identify their most frequent search topics. Respondents could select multiple options, so percentages total more than 100%. The data show that they primarily turned to AI for day-to-day nutritional optimization. More than half of the mothers searched for information on child growth supplements (237/442, 53.6%) and the nutritional value of various foods (235/442, 53.2%). A full breakdown of these search topics is available in Multimedia Appendix 4.

#### Measurement Model Evaluation

The measurement model was evaluated by examining indicator reliability, construct reliability, convergent validity, and discriminant validity. Indicator reliability was assessed through outer loadings, which ranged from 0.739 to 0.918, indicating that the majority of indicators adequately represent their respective constructs. Construct reliability was examined using Cronbach α and CR (rho_c). Cronbach α values ranged from 0.833 to 0.916, and rho_c values ranged from 0.882 to 0.941, all exceeding the recommended threshold of 0.70, demonstrating acceptable internal consistency. Convergent validity was evaluated through the average variance extracted, which ranged from 0.600 to 0.800, suggesting that more than half of the variance of each indicator was explained by the corresponding construct. These results indicate that the instrument demonstrates sufficient reliability and validity. Detailed values for each construct are presented in Table 4.

**Table 4.** Construct validity and reliability results. Indicator codes NFP1–NFP5 correspond to FPQ1-FPQ5 in the SmartPLS output (Figure 3), which retains the original instrument coding.

Variable and code	Indicator	OL^a^
AI^b^ Algorithmic Trust (AT)^c^		
AT1	I feel this AI provides pretty consistent nutritional advice for my child.	0.844
AT2	I believe this AI algorithm has accurate processing capabilities, thus minimizing errors in child nutrition recommendations.	0.875
AT3	I believe this algorithm was designed objectively without bias.	0.802
AT4	I know that the AI I use is not directed towards hidden commercial interests (eg, promotion of a particular brand).	0.764
AT5	I feel that this AI’s advice aligns with my needs as a mother in managing my child’s nutrition.	0.827
AI Health Information Quality (IQ)^d^		
IQ1	I consider the nutritional information provided by this AI to be accurate according to credible health references.	0.743
IQ2	The answers given by AI regarding children’s nutrition are complete and comprehensive.	0.763
IQ3	The menu suggestions provided by AI are appropriate to my child’s age and specific needs.	0.845
IQ4	Information from AI is presented in language that is easy for mothers like me to understand.	0.825
IQ5	I think the information from this AI is not contaminated by certain political interests.	0.825
AI Personalization Fit (PF**)** Perception^e^		
PF1	I feel that the food recommendations from AI are appropriate for my child’s growth stage.	0.805
PF2	I feel that the food suggestions from AI are quite in line with my child’s appetite.	0.802
PF3	I feel that the AI’s grocery suggestions are still within my family’s spending power.	0.789
PF4	I feel that I can implement the AI’s suggestions in the midst of my daily activities.	0.761
PF5	I feel that the AI’s suggestions are in line with my child’s physical needs.	0.861
AI Information-Seeking Intensity (ISI)^f^		
ISI1	I often use AI applications to get the information I need.	0.836
ISI2	I often use AI to check information from other sources.	0.846
ISI3	I often ask AI if I need ideas for doing something.	0.850
ISI4	I usually ask questions over and over again so that I really understand the information I need.	0.758
AI Social Proof (SP) Sensitivity^g^		
SP1	I felt more confident in AI when I learned that many other mothers were using it too.	0.878
SP2	Knowing that this app is used by so many people makes me even more confident in the accuracy of this AI.	0.918
SP3	I tend to choose AI that is popular among mothers because I believe that “the more users, the less error in the AI.”	0.893
SP4	If the majority of mothers share positive stories about an AI, then I will be more likely to believe in this AI.	0.887
Cognitive Empowerment (CE) in Health Decision-Making^h^		
CE1	This AI encouraged me to learn to understand the nutritional terms needed in child nutrition planning.	0.812
CE2	Using AI has made me understand better how to measure children’s nutritional content.	0.880
CE3	It became easier for me to conclude the information provided by this AI through the explanations given in a coherent manner.	0.846
CE4	I have become better able to sort out information from AI that is useful for addressing my child’s nutritional issues.	0.861
CE5	I was taught by AI to be more independent when making decisions about managing my child’s nutrition.	0.808
Parental Self-Compassion (PSC) in Feeding Context^i^		
PSC1	I can stay calm when my child’s meal plan doesn’t go as expected.	0.808
PSC2	I feel more at ease knowing that toddler food refusal behavior is a common experience among mothers, not a reflection of my own parenting failure.	0.820
PSC3	I don’t blame myself when my child refuses to eat the healthy food I have prepared.	0.817
PSC4	I have learned to manage my stress response when my child refuses to eat during mealtimes.	0.750
PSC5	I don’t beat myself up too much when my kids’ food choices don’t always go according to plan.	0.783
eHealth Self-Efficacy (ESE)^j^		
ESE1	I am confident using AI to help me solve my child’s eating problems.	0.797
ESE2	I am confident that I can find other reliable sources of information if the AI’s advice on children’s diet does not suit my child’s condition.	0.844
ESE3	I am confident that I can find a variety of menus that suit my child’s nutritional needs through appropriate AI prompting (giving instructions or questions).	0.813
ESE4	I am confident that I can ask or give orders to the AI myself without the help of others.	0.842
Perceived Nutrition-Oriented Feeding Practices (NFP)^k^		
NFP1	I can usually recognize the signs of fullness in my child correctly.	0.752
NFP2	Generally, I have consistently implemented a regular schedule for main meals and snacks.	0.790
NFP3	I am able to limit giving my child less nutritious food.	0.753
NFP4	I usually think about what kind of food is healthy enough for my child every day.	0.835
NFP5	I always read the nutritional content on the packaging of food prepared for my child.	0.739

a OL: Outer loading.

b AI: artificial intelligence.

c Mean=3.7873, Cronbach α=0.881, Rho_a=0.884, Rho_c=0.913, and average variance extracted=0.678.

d Mean=3.9090, Cronbach α=0.860, Rho_a=0.860, Rho_c=0.899, and average variance extracted =0.642

e Mean=3.7805, Cronbach α=0.863, Rho_a=0.868, Rho_c=0.901, and average variance extracted=0.647

f Mean=3.9745, Cronbach α=0.841, Rho_a=0.842, Rho_c=0.894, and average variance extracted=0.678

g Mean=3.5249, Cronbach α=0.916, Rho_a=0.918, Rho_c=0.941, and average variance extracted=0.800

h Mean=3.8181, Cronbach α=0.897, Rho_a=0.898, Rho_c=0.924, and average variance extracted=0.709

i Mean=3.8443, Cronbach α=0.855, Rho_a=0.855, Rho_c=0.896, and average variance extracted=0.633

j Mean=3.7755, Cronbach α=0.843, Rho_a=0.846, Rho_c=0.894, and average variance extracted=0.679

k Mean=4.0443, Cronbach α=0.833, Rho_a=0.835, Rho_c=0.882, and average variance extracted=0.600

Discriminant validity was rigorously evaluated using the HTMT ratio criteria, which can be seen in the Multimedia Appendix 5. The analysis results indicate that all constructs have adequately met the discriminant validity criteria. The research data recorded the highest HTMT ratio of 0.922 in the relationship between the AI Algorithmic Trust (AT) construct and Parental Self-Compassion in Feeding Context (PSC). This study then applied further testing in the form of an HTMT inference test to evaluate the high ratio in this relationship. The test results proved that the upper limit of the 90% confidence interval (bias-corrected) was at 0.955. This result supports the empirical distinctiveness between constructs, as the upper confidence bound falls below the absolute threshold of 1.000, indicating that all constructs in this study meet the discriminant validity criteria.

#### Structural Model and Hypothesis Testing

Before evaluating the structural paths, the model was assessed for multicollinearity and potential CMB. Following the full collinearity assessment approach proposed by Kock [67], the inner VIF was examined for all predictor constructs. As shown in Multimedia Appendix 6, all inner VIF values ranged from 1.376 to 2.785. Since these values fall below the conservative threshold of 3.3, the predictor constructs can be considered empirically independent, and CMB is unlikely to be a serious concern in this dataset

**Figure 3.**
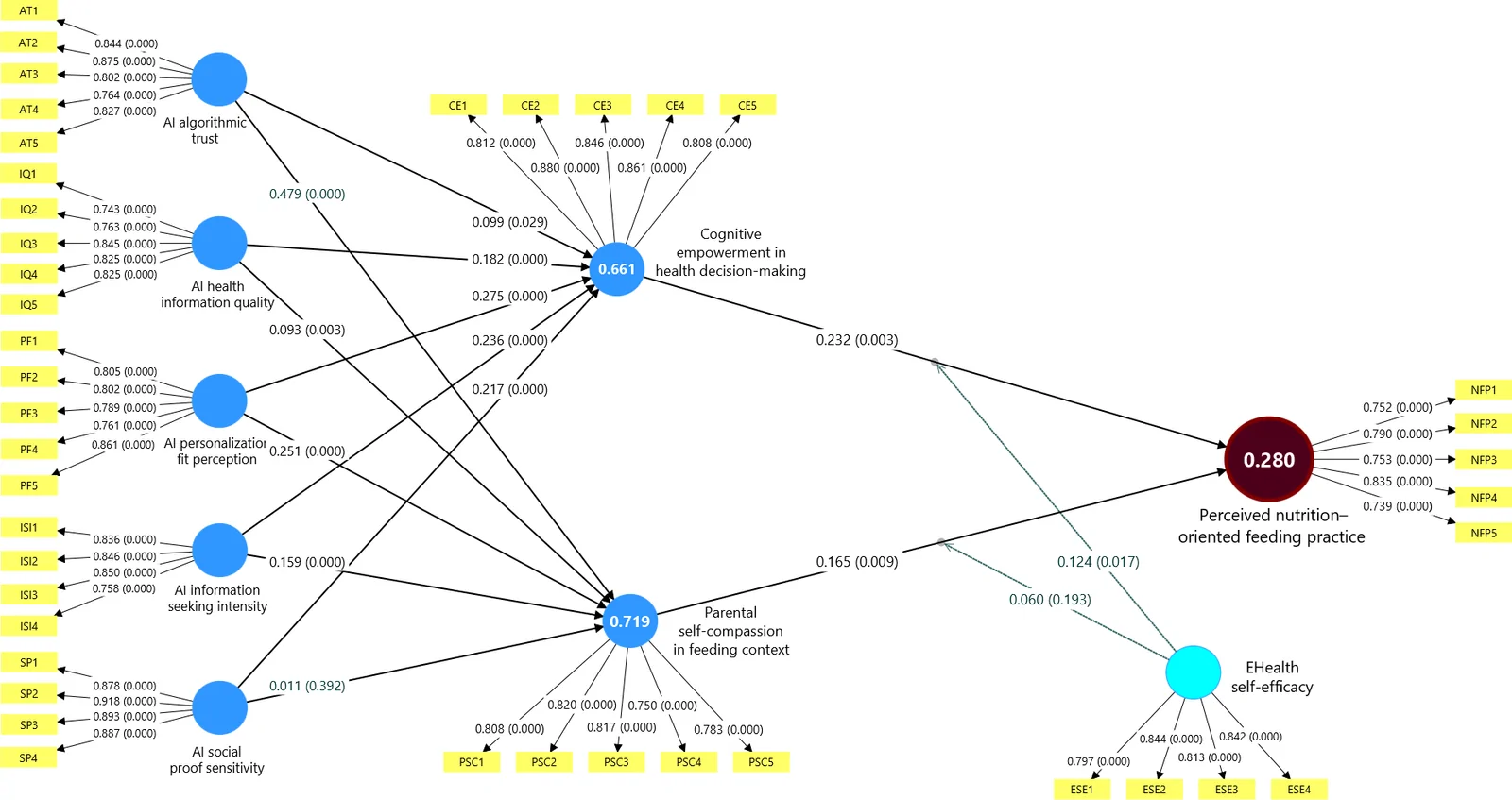
Structural (inner) model estimated with PLS-SEM. Circles represent latent constructs and yellow rectangles their measured indicators. Light blue circles denote the exogenous predictors and the 2 mediators; the cyan circle denotes eHealth self-efficacy, modeled as a moderator (interaction effects shown as dashed teal arrows); and the dark red circle denotes the endogenous target construct. Arrow values are path coefficients with P values within parentheses, and values within the endogenous circles are *R*² values. AI: artificial intelligence; PLS-SEM: partial least squares–structural equation modeling.

Based on the out-of-sample predictive power evaluation using PLSpredict, all latent variables yielded Q²_predict_ values greater than zero: Cognitive Empowerment in Health Decision-Making (0.992), Parental Self-Compassion in Feeding Context (0.994), and Perceived Nutrition-Oriented Feeding Practices (0.989)—indicating low descriptive prediction error at the construct level. These values, while notably high, are consistent with the strong construct reliability observed in the measurement model (Cronbach α 0.833‐0.916, CR 0.882‐0.941) and the large sample size (N=442), both of which enhance out-of-sample predictive stability [68]. Because Q²_predict_ is a descriptive metric based on error reduction, predictive validity was further examined through the CVPAT, which compares model performance against 2 benchmarks of increasing stringency: the naive indicator average (IA) and the stricter linear model (LM). Against IA, the model produced significant improvements across all constructs (*P*<.001). Against LM, only the overall row simultaneously produced a negative average loss difference and a significant *P* value of .024—a combination that under CVPAT constitutes formal predictive validity at the aggregate model level. Future replications should interpret construct-level predictive advantages with caution. Following this validation, the complete structural model, detailing the path coefficients and variance explained, is illustrated in Figure 3.

The CVPAT was used to evaluate the model’s out-of-sample predictive capabilities. As shown in Table 5, the PLS-SEM produced significantly lower prediction errors than the naive IA benchmark across all constructs and the overall model (*P*<.001). When compared against the more conservative LM benchmark, the overall model demonstrated a significantly negative average loss difference (*P*=.02), although individual constructs (CE and PSC) did not reach statistical significance against this benchmark. Based on the overall model’s significantly lower prediction errors relative to both the IA and LM benchmarks, the model demonstrates adequate predictive validity at the aggregate level.

**Table 5.** Cross-Validated Predictive Ability Test.

Variable	PLS-SEM^a^ versus IA^b^				PLS-SEM versus LM^c^			
	PLS loss	IA loss	Average loss difference	*P* value	PLS loss	LM loss	Average loss difference	*P* value
CE^d^	0.280	0.515	−0.235	<.001	0.280	0.287	−0.007	.30
PSC^e^	0.273	0.496	−0.223	<.001	0.273	0.276	−0.002	.62
NFP^f^	0.364	0.430	−0.066	<.001	0.364	0.384	−0.020	.05
Overall	0.305	0.480	−0.175	<.001	0.305	0.315	−0.010	.02

a PLS-SEM: partial least squares–structural equation modeling.

b IA: indicator average.

c LM: linear model.

d CE: cognitive empowerment in health decision-making.

e PSC: parental self-compassion in feeding context.

f NFP: perceived nutrition-oriented feeding practices.

Of the 14 hypotheses tested, 12 were supported, while 2 (H10 and H14) were not supported as zero fell within their confidence interval ranges. This indicates insufficient evidence that AI Social Proof Sensitivity is associated with Parental Self-Compassion (H10), or that eHealth Self-Efficacy significantly moderates the relationship between Parental Self-Compassion and Perceived Nutrition-Oriented Feeding Practices (H14). Among the independent variables, AI Algorithmic Trust showed the strongest association with Parental Self-Compassion (*β*=.479; *P*<.001), while AI Personalization Fit Perception showed the strongest association with Cognitive Empowerment (*β*=.275; *P*<.001). Cognitive Empowerment demonstrated a stronger association with Perceived Nutrition-Oriented Feeding Practices (*β*=.232; *P*=.003) than did Parental Self-Compassion (*β*=.165; *P*=.009). For the moderation analysis, eHealth Self-Efficacy significantly strengthened the pathway from Cognitive Empowerment to Perceived Nutrition-Oriented Feeding Practices (*β*=.124; *P*=.02). The predominance of small *f*² values reflects the model’s indirect association architecture rather than weak associations. Because 5 correlated AI engagement predictors converge on each intervening construct, the incremental variance of any single path is necessarily diluted, even as the collective *R*² remains substantial (0.661; 0.719). The negligible effects (H1, *f*²=0.011; H12, *f*²=0.017; and H13, *f*²=0.018) operate reliably in the hypothesized direction while adding little unique variance once stronger predictors are accounted for; this pattern also applies to H13, given that interaction terms are typically smaller than main effects in behavioral models [69]. The single medium effect, AI Algorithmic Trust → Parental Self-Compassion (H2, *f*²=0.293), identifies the emotional trust pathway as the model’s most prominent association, consistent with the difficulty of explaining feeding outcomes from psychosocial predictors alone. A bootstrap MGA comparing mothers of children aged 6‐24 months (n=136) and 25‐59 months (n=306) found no statistically significant differences across any of the structural paths (all *P*>.05; smallest difference *P*=.13), indicating that the modeled associations were stable across this age range (Multimedia Appendix 3). The complete hypothesis testing results are presented in Table 6.

**Table 6.** Hypothesis testing results.

Hypotheses	*β* ^a^	*P* value	Confidence interval		*f*^2^ value	Decision
			5.0%	95.0%		
H1: AI Algorithmic Trust → Cognitive Empowerment in Health Decision-Making	.099	.03	0.014	0.186	0.011	Hypothesis supported
H2: AI Algorithmic Trust → Parental Self-Compassion in Feeding Context	.479	<.001	0.397	0.558	0.293	Hypothesis supported
H3: AI Health Information Quality → Cognitive Empowerment in Health Decision-Making	.182	<.001	0.108	0.256	0.071	Hypothesis supported
H4: AI Health Information Quality → Parental Self-Compassion in Feeding Context	.093	.003	0.036	0.146	0.023	Hypothesis supported
H5: AI Personalization Fit Perception → Cognitive Empowerment in Health Decision-Making	.275	<.001	0.189	0.361	0.083	Hypothesis supported
H6: AI Personalization Fit Perception → Parental Self-Compassion in Feeding Context	.251	<.001	0.176	0.326	0.084	Hypothesis supported
H7: AI Information Seeking Intensity → Cognitive Empowerment in Health Decision-Making	.236	<.001	0.17	0.301	0.099	Hypothesis supported
H8: AI Information-Seeking Intensity → Parental Self-Compassion in Feeding Context	.159	<.001	0.093	0.226	0.054	Hypothesis supported
H9: AI Social Proof Sensitivity → Cognitive Empowerment in Health Decision-Making	.217	<.001	0.123	0.309	0.057	Hypothesis supported
H10: AI Social Proof Sensitivity → Parental Self-Compassion in Feeding Context	.011	.39	−0.057	0.073	0	Hypothesis not supported
H11: Cognitive Empowerment in Health Decision-Making → Perceived Nutrition-Oriented Feeding Practices	.232	.003	0.097	0.367	0.028	Hypothesis supported
H12: Parental Self-Compassion in Feeding Context → Perceived Nutrition-Oriented Feeding Practices	.165	.009	0.044	0.275	0.017	Hypothesis supported
H13: eHealth Self-Efficacy × Cognitive Empowerment in Health Decision-Making → Perceived Nutrition-Oriented Feeding Practices	.124	.02	0.029	0.216	0.018	Hypothesis supported
H14: eHealth Self-Efficacy × Parental Self-Compassion in Feeding Context → Perceived Nutrition-Oriented Feeding Practices	.06	.19	−0.052	0.174	0.003	Hypothesis not supported

a*β* : path coefficient.

#### Advanced Analytics

**Figure 4.**
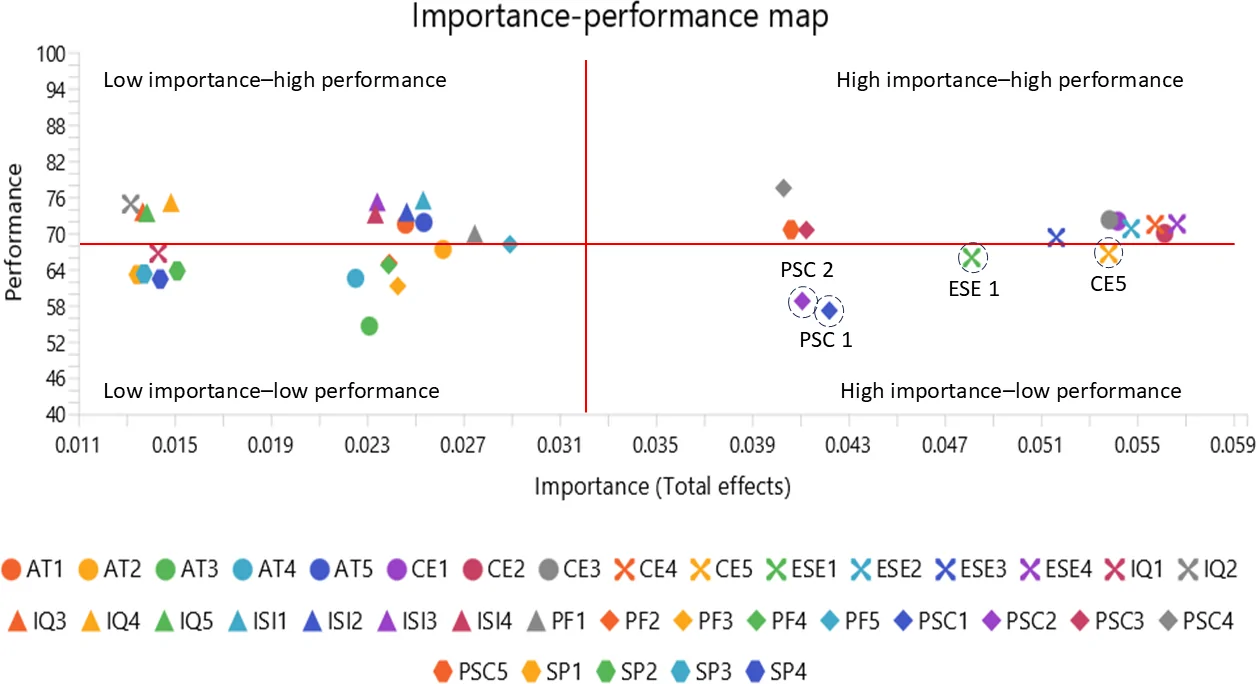
Importance-Performance Map Analysis indicators. CE: cognitive empowerment; ESE: eHealth self-efficacy; PSC: parental self-compassion.

AI engagement constructs were related to perceived nutrition-oriented feeding practices via both intervening constructs. Nine of 10 specific indirect effects and all 5 total indirect effects were supported, with the strongest indirect association via cognitive empowerment. The pathway from AI social proof sensitivity through parental self-compassion was not supported, consistent with H10. As the model included no direct paths, these are reported as indirect associations; because no direct paths were estimated, mediation is not inferred. Full results are reported in Multimedia Appendix 7. In addition, this study also applies IPMA to evaluate intervention priorities practically (Figure 4).

The IPMA identified 4 indicators positioned in the high-importance, low-performance quadrant, signaling priority areas for future intervention development. PSC1 (staying calm when meal plans do not go as expected) and PSC2 (feeling at ease knowing toddler food refusal is a common experience) both reflect the emotional regulation dimension of parental self-compassion—suggesting that AI-assisted feeding tools may need to incorporate emotional support and normalization features rather than focusing solely on informational content. ESE1 (confidence in using AI to solve child eating problems) indicates that user confidence in AI-based feeding solutions is a key gap, pointing to the need for progressive skill-building and user onboarding features. CE5 (learning to be independent in nutrition decisions through AI) suggests that AI platforms should scaffold—rather than replace—maternal decision-making autonomy. Together, these indicators suggest that future AI-assisted feeding tools should prioritize emotional support, confidence-building, and decision autonomy alongside informational accuracy. The mapping results showed that the data distribution placed indicators of parental self-compassion (PSC1 and PSC2), eHealth self-efficacy (ESE1), and cognitive empowerment (CE5) in areas of high importance with low performance. These findings may help identify potential priorities for future intervention development.

## Discussion

### Principal Findings

This mixed methods study investigated how urban Indonesian Alpha Mothers, a digitally literate, highly educated, and evidence-driven caregiver profile, had an increasing engagement with generative AI as a decision support partner in toddler feeding. Beyond the structural model, the qualitative phase contextually refined 5 literature-informed AI engagement dimensions relevant to maternal AI-mediated toddler feeding—algorithmic trust, health information quality, personalization fit perception, information-seeking intensity, and social proof sensitivity. Although these dimensions align with existing constructs in the literature, their context-specific refinement through mothers’ lived experiences and their integration into a single framework for this specific context represent the study’s principal qualitative contribution. The quantitative outcome examined was maternal perception of nutrition-oriented feeding practices and not observed feeding behavior or child nutrition outcomes. The findings suggest that associations between AI engagement and perceived nutrition-oriented feeding practices may involve 2 distinct indirect associations: 1 cognitive and 1 emotional. Mothers who trusted AI and found its recommendations relevant to their child’s feeding context were better able to evaluate and adapt its suggestions on their own terms. Mothers who perceived AI as nonjudgmental and objective reported lower maternal guilt. In this study, it is captured as parental self-compassion. Both pathways were independently associated with better perceived nutrition-oriented feeding practices, although the cognitive pathway carried greater weight.

Four findings warrant closer attention. AI algorithmic trust was the strongest predictor of parental self-compassion, suggesting that lower emotional burden may be more closely associated with trust in the AI system rather than with its popularity. AI personalization fit perception was the strongest predictor of cognitive empowerment, more so than algorithmic trust, information quality, or information-seeking intensity. Alpha Mothers do not appear to value accurate AI content alone. The findings suggest that they also value advice tailored to their child, daily routines, and cultural feeding practices. Beyond personalization, AI social proof sensitivity predicted cognitive empowerment but had no bearing on parental self-compassion. Community support, whether in the form of ratings, peer recommendations, or popularity signals, may shape a mother’s opinion about AI. Yet, it will not affect her inner convictions as a parent. Self-compassion in this context comes from personal experience and not from external reassurance.

Finally, eHealth self-efficacy strengthened the pathway from cognitive empowerment to nutrition-oriented feeding practices. However, it played no moderating role in the self-compassion pathway. Digital confidence and emotional self-regulation appear to be separate capacities that call for separate intervention approaches. The findings suggest that AI may serve as a supportive tool that complements, rather than replaces, maternal judgment and professional nutrition counseling.

### Comparison With Prior Work

Algorithmic trust emerged as the strongest predictor of parental self-compassion in this study. In the digital health literature, trust in AI has largely been treated as a functional matter, assessed through criteria of technical performance, accuracy, and system credibility [70]. Less attention has been paid, however, to whether trust in AI also carries an emotional function. Recent empirical work suggests that patients’ experiences with AI health systems extend well beyond rational cost-benefit evaluation, with emotional responses such as anxiety, cognitive overload, and resistance playing a significant role in how users actually engage with these tools [71,72]. The concept of algorithmic authority provides a framework for interpreting this emotional dimension.

The present findings add a maternal caregiving dimension to this picture. For these urban Indonesian mothers, trusting an AI system was not simply a matter of judging whether its nutritional advice was clinically sound. It also determined whether that trust could absorb some of the guilt and cognitive load that accompanies daily toddler-feeding decisions. AI-driven pediatric tools may benefit from being designed with this emotional function in mind, not only to improve informational output but also to offer meaningful psychological relief to the caregivers who rely on them.

Trust in health AI has been shown to rest less on technical performance than on the degree to which a system reflects the user’s own values and ways of knowing [73]. For the Alpha Mothers in this study, factual accuracy was merely a baseline. Qualitative findings suggest that trust was closely tied to perceptions of commercial neutrality and value alignment.

The result indicated that AI personalization fit perception was the strongest predictor of cognitive empowerment compared with the other 4 variables. The dominance of AI personalization as a predictor of cognitive empowerment in these findings aligns with existing literature on parental digital behavior. This pattern is consistent with findings that parents of preschool children prioritize family-adapted technology over standardized content [57,74,75]. The findings suggest that mothers in this study preferred highly contextualized nutritional guidance. This finding is consistent with the interpretation that for this demographic, personalization is the strongest predictor associated with cognitive empowerment.

However, this study structural model also reveals that this technology-driven empowerment has distinct boundaries, particularly concerning the emotional complexities of parenting. Specifically, the rejection of the hypotheses regarding AI Social Proof Sensitivity (H10) and the moderating role of eHealth Self-Efficacy (H14) indicates that while AI can cognitively empower mothers, technical and social features were not associated with higher levels of Parental Self-Compassion in this study. Knowing that other mothers use AI (social proof) or possessing high technical skills to navigate health information (eHealth self-efficacy) does not organically translate into reduced maternal guilt or enhanced emotional grace when facing feeding challenges.

The nonsignificant relationship between AI social proof sensitivity and parental self-compassion may reflect a dynamic specific to digital parenting environments. When mothers evaluate AI guidance through the lens of social proof, they are simultaneously exposed to a wide and often contradictory range of parenting standards. Far from offering reassurance, this collective validation tends to fuel upward social comparison and maternal competition. Recent empirical research indicates that exposure to heterogeneous parenting norms in digital spaces intensifies the internalization of an unrealistic “good mother” ideology [18,76]. True self-compassion requires self-kindness and the acceptance that all parents struggle. However, this mindset is incredibly hard to maintain in digital spaces that constantly push idealized standards, leaving mothers feeling like they fall short. High sensitivity to social proof, in this reading, does not appear to soften self-judgment and may instead be associated with greater self-criticism.

eHealth self-efficacy moderated the cognitive empowerment pathway but not the pathway from parental self-compassion to nutrition-oriented feeding practices. Digital self-efficacy has been shown to associate positively with health self-management behaviors through chain mediation in general population samples [77]. The divergence in these findings reflects the nature of self-compassion itself. Cognitive empowerment involves active information processing and responds to greater digital confidence. Self-compassion, by contrast, is an affective response rooted in self-acceptance. A mother’s ability to navigate health platforms does not make her more forgiving of herself when feeding goes wrong. These 2 observed patterns may reflect differences in the underlying psychological constructs and require different intervention approaches. Ultimately, these different paths lead to the question: what can a model focused on AI engagement realistically explain?

Taken together, these findings can be interpreted through the algorithmic authority lens introduced earlier [32]. This interpretation is offered as a theoretical reading of the cross-sectional associations observed in this study rather than as a directly tested causal account. The strongest pathway, from AI algorithmic trust to parental self-compassion, may be consistent with early-stage algorithmic authority formation, in which higher trust is associated with reliance on AI not only for information but also for emotional reassurance. Within this theoretical framing, such reliance could mark the point at which AI begins to substitute for, rather than support, maternal judgment, although the present data cannot establish whether this substitution actually occurs. The nonsignificant association for H10 refines this picture: in maternal caregiving, awareness that other mothers use AI was not associated with parental self-compassion, a pattern interpreted here as peer signals inviting social comparison rather than reassurance [18,76]. The moderation finding adds further insight: eHealth self-efficacy strengthened only the cognitive pathway, suggesting that digital literacy helps mothers think critically about AI advice but does not protect them from becoming emotionally dependent on it.

Together, these patterns may suggest, at a theoretical level, that conditions for algorithmic authority are most pronounced when high trust in AI coexists with low emotional resilience, the pattern the AI parenting nutrition alignment approach is intended to address. This raises a conceptual concern about potential algorithmic dependency: if high trust in AI and emotional relief through its use were to develop together, mothers might progressively reduce consultations with health care professionals, perceiving clinical guidance as less necessary [32,42]. Consequently, AI-generated inaccuracies may remain undetected, potentially delaying appropriate referral when a child presents with genuine nutritional concerns. In the toddler-feeding domain specifically, these risks take concrete forms: AI-generated advice may recommend age-inappropriate foods, overlook individual allergies, or suggest incorrect nutrient dosages [28], concerns corroborated by qualitative participants who reported receiving recommendations containing potentially harmful ingredients for their child’s condition. Because detecting such errors depended on the mother’s own vigilance, the risk was unevenly distributed—least for mothers with stronger nutritional knowledge and critical appraisal skills, and greatest for those most reliant on AI. This remains a hypothesized risk that the cross-sectional design cannot confirm and that warrants longitudinal investigation.

The effect size pattern warrants careful interpretation. AI Algorithmic Trust showed the strongest contribution to Parental Self-Compassion, while the other supported pathways showed smaller contributions. Some pathways, although statistically significant, contributed only modestly to the outcome. Two factors help explain this pattern. First, nutrition-oriented feeding practices are shaped by many caregiving factors, such as economic, cultural, situational, and child-specific factors that fall outside any AI engagement model. Second, moderation effects are typically smaller than main effects in behavioral models [69]. The findings should therefore be read as statistically robust but practically modest contributions of AI engagement to perceived nutrition-oriented feeding practices, rather than as evidence that AI engagement is the dominant driver of nutrition-oriented feeding practices. The same reality is reflected in the modest overall explanatory power for nutrition-oriented feeding practices. This suggests that AI engagement, together with the 2 intervening constructs, captures only part of what shapes feeding practices, with substantial remaining variance attributable to factors outside the model, such as socioeconomic conditions, family food culture, child temperament, maternal mental health, and situational demands. Furthermore, perceived measures of caregiving behavior—as opposed to directly observed practices—are known to be shaped by momentary contextual influences and subjective interpretation, which further constrains the variance explainable by any structural predictor set. Structural models targeting behavioral outcomes rarely explain more than a small proportion of variance, even when theory-driven and methodologically robust. The identification of algorithmic trust and parental self-compassion as significant psychosocial pathways therefore represents a meaningful contribution within this explanatory context. The perceived nutrition-oriented feeding practices construct in this model captures the nutritional and structural dimensions of infant and young child feeding guidance, namely, schedule consistency, nutritional appraisal, and limitation of low-nutrient foods, together with attentiveness to satiety signals. It does not operationalize the reciprocal responsiveness components central to the WHO or UNICEF definition of responsive feeding, which additionally require assessment of coercive feeding pressure and child autonomy support [5,6]. While the findings suggest that AI engagement may reinforce these nutrition-oriented practices via its association with cognitive empowerment, current AI platforms remain unbenchmarked against WHO or UNICEF responsive feeding frameworks or the structured competencies delivered through the UNICEF C-IYCF Counselling Package [25]. AI-assisted feeding support should therefore complement, not replace, established C-IYCF counseling models.

### Implications

The following implications should be interpreted as preliminary directions derived from cross-sectional associations rather than as established prescriptions. The policy, clinical practice, and system development levels outlined in this section each warrant validation through longitudinal investigation, intervention trials, and contextual adaptation prior to large-scale implementation. At the policy level, these findings highlight the growing importance of governance frameworks for AI-assisted parenting and nutrition support. As generative AI becomes increasingly integrated into health care, international guidance has emphasized transparency, accountability, safety, and human oversight in AI-enabled health systems [78,79]. Indonesia has similarly established foundational principles for responsible AI development through Ministerial Circular No. 9/2023 [80]. However, no dedicated framework currently addresses AI-generated child nutrition advice or clearly defines accountability when such advice is associated with harm. Future policy efforts may therefore benefit from establishing standards for transparency, evidence attribution, and alignment with WHO and UNICEF responsive feeding guidance, while ensuring that AI-assisted nutrition support complements rather than replaces professional nutrition counseling.

At the clinical practice level, the findings suggest that pediatricians, nutritionists, and public health practitioners may increasingly serve as facilitators who help mothers critically evaluate AI-generated nutrition advice and integrate it with professional guidance and their child’s individual circumstances. Within the Indonesian context, future research may explore whether AI literacy and nutrition counseling can be incorporated into existing maternal and child health services, including Posyandu and routine well-child care. Such approaches should position AI as a complement to, and not a substitute for, professional nutrition counseling while strengthening maternal cognitive empowerment and critical appraisal skills.

At the system development level, the findings provide preliminary insights that may inform future AI-assisted feeding support tools. The AI Parenting Nutrition Alignment Approach highlights the potential value of contextualized guidance, transparent explanations, and support for critical evaluation of AI-generated recommendations. Future systems may also benefit from benchmarking against WHO and UNICEF responsive feeding guidance to improve consistency with evidence-based nutrition recommendations. Development and implementation of such tools would likely require collaboration among pediatricians, nutritionists, public health practitioners, AI developers, and policymakers to ensure appropriate clinical oversight, safety, and governance. However, these implications remain conceptual and require evaluation through longitudinal and intervention-based research before broader implementation.

### Limitations

Several limitations should be acknowledged. First, the sample was drawn from a specific demographic profile—urban, digitally active mothers who actively use generative AI—referred to in this study using the descriptive label “Alpha Mother.” This label functions as a recruitment descriptor rather than a validated theoretical construct, and findings should not be generalized beyond this defined demographic profile. Whether the patterns observed here apply to mothers from different socioeconomic, educational, or digital engagement profiles remains an open empirical question. Second, recruitment through digital parenting communities likely introduced self-selection bias toward mothers already favorably disposed toward generative AI. Findings should therefore be interpreted as reflecting the experiences of digitally active, AI-engaged urban mothers rather than urban Indonesian mothers more broadly.

Third, all data relied on self-report. Mothers may have overstated their trust in AI or their sense of self-compassion to align with perceived parenting ideals, particularly given the social pressures that surround toddler feeding in urban Indonesian contexts. Furthermore, perceived nutrition-oriented feeding practices capture maternal self-assessment, not observed feeding behavior or objective child nutrition outcomes. The instrument assesses nutrition-focused and structural aspects of feeding; coercive feeding pressure, caregiver responsiveness to the full range of hunger and satiety cues, and child autonomy support were not measured. Findings therefore speak to the nutritional and structural dimensions of feeding rather than to caregiver-child interactional quality and should not be read as evidence regarding responsive feeding as defined by WHO and UNICEF. Future work should triangulate this construct against behavioral instruments such as the Comprehensive Feeding Practices Questionnaire and against anthropometric indicators.

Fourth, the cross-sectional design and the modest variance explained limit interpretation. The associations identified through PLS-SEM are statistically robust but cannot establish whether AI engagement changes maternal behavior over time, and the low explained variance indicates that AI engagement is only one of several factors shaping feeding practices. The design also permits alternative explanations the present data cannot adjudicate: the trust-outcome relationship may be reversed or reciprocal, as mothers who already possess greater feeding confidence, knowledge, or emotional resilience may be more predisposed to trust AI and report favorable feeding experiences. An unmeasured third variable, such as general health literacy or socioeconomic advantage, could likewise elevate both, and such confounding is difficult to exclude given the self-selected sample. The reported associations should therefore be read as compatible with, but not confirmatory of, the mechanistic interpretation advanced in the Principal Findings section. Improvements in perceived nutrition-oriented feeding practices should therefore not be interpreted as evidence of improved child nutritional or developmental outcomes. In addition, although the sample spanned a broad age range (6‐59 months), an MGA confirmed that the structural estimates did not differ significantly between younger and older children (Multimedia Appendix 3); the younger stratum nonetheless follows the survey’s 6‐ to 24-month category, 1 month wider than the WHO 6‐ to 23-month cutoff, and its smaller size warrants caution in interpreting subgroup estimates.

Fifth, social desirability and CMB cannot be fully excluded, as all constructs were self-reported through a single instrument at one time point. Although procedural remedies and a full collinearity assessment (all inner VIF <3.3) indicate that CMB is unlikely to be severe, these safeguards reduce rather than eliminate the risk. In addition, the 5 AI engagement dimensions were newly developed here and have not been externally validated, and the AI parenting nutrition alignment approach that organizes them adapts an existing framework [44] not yet validated in this domain. Findings involving them should be regarded as preliminary.

Finally, this study did not independently audit the clinical accuracy of AI-generated nutritional advice received by participants; the constructs measured reflect mothers’ perceptions of AI quality rather than objective evaluation of AI output. Future research should systematically benchmark AI-generated feeding advice against WHO or UNICEF responsive feeding recommendations and the C-IYCF counseling competency framework, a critical step toward developing clinically validated AI-assisted feeding platforms.

### Conclusions

This mixed methods study suggests that generative AI is not independently associated with perceived nutrition-oriented feeding practices. Associations between AI engagement and perceived nutrition-oriented feeding practices were strongest when mothers trusted the system and perceived its recommendations as fitting their child and household realities. Two distinct indirect associations may help describe these associations. Algorithmic trust was associated with parental self-compassion, which was linked to lower emotional burden during feeding challenges, whereas personalization fit was associated with cognitive empowerment, which in turn was associated with mothers’ self-reported capacity to critically evaluate and adapt AI advice.

These indirect associations may reflect potentially important constructs by which AI engagement is associated with perceived nutrition-oriented feeding practices. Within the data, the perceived value of AI in toddler feeding appeared to relate less to algorithmic sophistication than to perceived trust, contextual fit, and support for maternal judgment. Beyond these associations, and extending past what the present cross-sectional data can establish, the findings may hold tentative implications for clinicians, policymakers, and developers. They may inform future research on, and the eventual design and evaluation of, AI-assisted feeding support, provided such tools are positioned to complement rather than supplant professional nutrition counseling. These implications remain hypotheses for future longitudinal and intervention research rather than evidence-based recommendations. In addition to these quantitative pathways, this study contributes a qualitative typology of AI engagement constructs specific to the toddler-feeding context, providing a conceptual foundation for future research at the intersection of AI, maternal cognition, and child nutrition.

## Supplementary material

10.2196/100061 Multimedia Appendix 1 Eligibility screening and verification procedures.

10.2196/100061 Multimedia Appendix 2 Audit trail phase 1.

10.2196/100061 Multimedia Appendix 3 Age-based multigroup analysis.

10.2196/100061 Multimedia Appendix 4 Table frequencies of artificial intelligence search topics.

10.2196/100061 Multimedia Appendix 5 Table discriminant validity (Heterotrait-Monotrait ratio).

10.2196/100061 Multimedia Appendix 6 Collinearity statistics (inner variance inflation factor values).

10.2196/100061 Multimedia Appendix 7 Indirect effects table.

## Abbreviations

C-IYCF: Community Infant and Young Child Feeding
CMB: common method bias
CR: composite reliability
CVPAT: Cross-Validated Predictive Ability Test
H: hypothesis
HTMT: Heterotrait-Monotrait
IA: indicator average
IPMA: Importance-Performance Map Analysis
LM: linear model
MGA: multigroup analysis
PLS-SEM: partial least squares–structural equation modeling
UNICEF: United Nations Children’s Fund
VIF: variance inflation factor
WHO: World Health Organization
