# Supplementary material for: Generative AI Engagement and Perceived Nutrition-Oriented Feeding Practices Among Urban Indonesian Mothers: Mixed Methods Study
Source: JMIR Pediatr Parent. 2026 Sep 21;9:e100061. doi: 10.2196/100061 (PMC13592562; doi:10.2196/100061)
Supplement: Supplementary file 1 [file pediatrics-v9-e100061-s001.docx]

**Multimedia Appendix 1**

**Eligibility Screening and Verification Procedures**

Participant eligibility was determined through a two-stage process: a brief pre-screening to identify candidates meeting two essential criteria, followed by verification of the remaining eligibility criteria through items embedded within the main survey and through manual confirmation by the research team during recruitment.

**Stage 1. Pre-screening items**

Administered prior to the main survey to identify eligible participants:

1. Do you currently have a child aged 6 to 59 months?

- Yes
- No

2. Have you used generative AI platforms (e.g., ChatGPT, Gemini, Grok, Claude, Copilot, Bing) to seek information about childcare or child nutrition?

- Yes
- No

*Participants answering "No" to either item were not invited to the main survey.*

**Stage 2. Eligibility verification items embedded in the main survey**

1. In which city do you currently reside?

- Greater Jakarta
- Bandung
- Surabaya
- Medan
- Other (specify): _______
  *Used to verify urban residence in the designated study regions.*

2. How long have you been using generative AI platforms to seek childcare or child nutrition information?

- Less than 6 months
- 6 months or more
  *Used to confirm minimum duration of AI use.*

3. How often do you use generative AI platforms to seek childcare or child nutrition information?

- Less than 3 times per week
- 3 times per week or more
  *Used to confirm minimum frequency of AI use.*

4. Informed consent

- have read the participant information sheet and voluntarily agree to participate in this study.

**Stage 3. Manual verification by the research team**

Exclusion of healthcare professionals was conducted manually by the first author (LNA) during recruitment. Potential participants were directly asked whether their current occupation involved providing clinical healthcare services, specifically as a physician, nurse, nutritionist, or dietitian. Individuals confirming any of these occupations were excluded prior to questionnaire distribution. This procedure complemented the general occupation categories captured within the main survey demographic items.

**Application of eligibility criteria during data cleaning**

Responses falling outside the defined eligibility criteria :

- Residence outside the designated urban regions
- AI use shorter than 6 months
- Frequency of AI use less than 3 times per week,
- Post-hoc identification of healthcare professional status

were excluded from the analytic sample during the data cleaning process described in the methods section.
