# Supplementary material for: Generative AI Engagement and Perceived Nutrition-Oriented Feeding Practices Among Urban Indonesian Mothers: Mixed Methods Study
Source: JMIR Pediatr Parent. 2026 Sep 21;9:e100061. doi: 10.2196/100061 (PMC13592562; doi:10.2196/100061)
Supplement: Supplementary file 2 [file pediatrics-v9-e100061-s003.docx]

**Multimedia Appendix 3. Age-Based Multi-Group Analysis (MGA)**

*Bootstrap multi-group analysis comparing mothers of children aged 6–24 months (Group 1, n = 136) and 25–59 months (Group 2, n = 306). Estimation used 10,000 bootstrap subsamples, two-tailed, at the .05 significance level, on the same structural model reported in the main analysis (N = 442). The younger stratum reflects the survey’s 6–24-month response option, one month wider than the WHO 6–23-month cut-off.*

**Table Group-specific path coefficients and between-group difference tests**

| **Structural path** | **β Group 1 (6–24 mo)** | **β Group 2 (25–59 mo)** | **Diff. (G1−G2)** | **p (2-tailed)** | **Sig. diff.?** |
| --- | --- | --- | --- | --- | --- |
| AI Algorithmic Trust → CE | −0.144 | −0.136 | −0.009 | .994 | No |
| AI Info Quality → CE | 0.299 | 0.178 | 0.122 | .831 | No |
| AI Personalization Fit → CE | 0.260 | 0.442 | −0.183 | .586 | No |
| AI Info-Seeking Intensity → CE | 0.237 | 0.297 | −0.060 | .710 | No |
| AI Social Proof → CE | 0.340 | 0.181 | 0.159 | .396 | No |
| AI Algorithmic Trust → PSC | −0.055 | −0.820 | 0.765 | .299 | No |
| AI Info Quality → PSC | −0.168 | 1.054 | −1.222 | .133 | No |
| AI Personalization Fit → PSC | 0.457 | 0.195 | 0.263 | .559 | No |
| AI Info-Seeking Intensity → PSC | 0.251 | −0.001 | 0.251 | .304 | No |
| AI Social Proof → PSC | 0.100 | 0.229 | −0.129 | .756 | No |
| Cognitive Empowerment → NFP | 0.417 | −0.114 | 0.531 | .472 | No |
| Parental Self-Compassion → NFP | 0.251 | 0.243 | 0.009 | .835 | No |
| E-Health Self-Efficacy → NFP | 0.118 | 0.563 | −0.445 | .590 | No |
| ESE × CE → NFP (moderation) | 0.477 | 0.211 | 0.266 | .692 | No |
| ESE × PSC → NFP (moderation) | −0.311 | −0.183 | −0.128 | .798 | No |

*Note. CE = cognitive empowerment; PSC = parental self-compassion; NFP = perceived nutrition-oriented feeding practices; ESE = e-health self-efficacy. “Sig. diff.?” indicates whether the between-group difference is significant at p < .05 (bootstrap MGA, two-tailed). No path showed a significant difference between age groups; the smallest difference p-value across all fifteen paths was .133.*

**Interpretation**

None of the fifteen structural paths differed significantly between the younger and older age strata, indicating that the modeled associations are stable across developmental stage and supporting interpretation of the outcome construct across the full 6–59-month range.

For the cognitive empowerment → NFP path, the group-specific coefficients differed in magnitude (β = 0.417 vs β = −0.114), but the between-group difference was not statistically significant (p = .472). Given the smaller size of the younger stratum (n = 136), which yields wider confidence intervals and less stable point estimates than the older stratum (n = 306), this divergence is best interpreted as small-sample instability rather than a genuine age-dependent effect. Group-specific estimates are reported here in full rather than difference p-values alone, so that this pattern can be evaluated directly.

Similarly, several group-specific coefficients for paths to parental self-compassion (e.g., AI Info Quality → PSC, β = 1.054 in the older stratum; AI Algorithmic Trust → PSC, β = −0.820) fell well outside the range of the pooled estimates, a pattern consistent with multicollinearity-driven instability when five correlated predictors are estimated in subgroups of this size. As with the CE → NFP divergence, no between-group difference reached statistical significance, and these point estimates are best interpreted as artifacts of subgroup sample limitations rather than substantive age effects.

Conceptually, the outcome construct measures nutrition-focused, structural feeding practices (e.g., meal scheduling, limiting low-nutrition foods, reading nutritional labels) rather than developmentally timed responsive-feeding behaviours. Such structural practices apply similarly across the 6–59-month range, which provides a substantive explanation for the observed stability.
