# Supplementary material for: Generative AI Engagement and Perceived Nutrition-Oriented Feeding Practices Among Urban Indonesian Mothers: Mixed Methods Study
Source: JMIR Pediatr Parent. 2026 Sep 21;9:e100061. doi: 10.2196/100061 (PMC13592562; doi:10.2196/100061)
Supplement: Supplementary file 3 [file pediatrics-v9-e100061-s004.docx]

**Multimedia Appendix 4**

**Table frequencies of AI search topics (N=442)**

| Searched topics | Participants, n (%) |
| --- | --- |
| Child growth and development supplements | 237 (53.6) |
| Nutritional value of food sources | 235 (53.2) |
| Balanced diet composition | 193 (43.7) |
| Specific food allergies or intolerances | 144 (32.6) |
| Signs of child malnutrition | 123 (27.8) |
| Stunting prevention | 104 (23.5) |
| Other health or general topics | 29 (6.6) |
