# Supplementary material for: Generative AI Engagement and Perceived Nutrition-Oriented Feeding Practices Among Urban Indonesian Mothers: Mixed Methods Study
Source: JMIR Pediatr Parent. 2026 Sep 21;9:e100061. doi: 10.2196/100061 (PMC13592562; doi:10.2196/100061)
Supplement: Supplementary file 4 [file pediatrics-v9-e100061-s005.docx]

**Multimedia Appendix 5**

**Table discriminant validity (HTMT ratio)**

| Constructs | IQ | ISI | PF | SP | AT | CE | ESE | PSC | FPQ | 1 | 2 |
| --- | --- | --- | --- | --- | --- | --- | --- | --- | --- | --- | --- |
| 1. IQ |  |  |  |  |  |  |  |  |  |  |  |
| 2. ISI | 0.478 CI (0.393; 0.557) |  |  |  |  |  |  |  |  |  |  |
| 3. PF | 0.566  CI (0.490; 0.633) | 0.633 CI (0.567; 0.694) |  |  |  |  |  |  |  |  |  |
| 4. SP | 0.480 CI (0.401; 0.550) | 0.640 CI (0.571; 0.701) | 0.766 CI (0.721; 0.807) |  |  |  |  |  |  |  |  |
| 5. AT | 0.477  CI (0.400; 0.549) | 0.655 CI (0.591; 0.711) | 0.845 CI (0.802; 0.883) | 0.794 CI (0.748; 0.833) |  |  |  |  |  |  |  |
| 6. CE | 0.621 CI (0.537; 0.692) | 0.734 CI (0.676; 0.785) | 0.807 CI (0.760; 0.849) | 0.757 CI (0.705; 0.801) | 0.748 CI (0.700; 0.792) |  |  |  |  |  |  |
| 7. ESE | 0.669 CI (0.588; 0.732) | 0.729 CI (0.662; 0.793) | 0.804 CI (0.760; 0.843) | 0.809 CI (0.754; 0.856) | 0.763 CI (0.712; 0.807) | 0.852 CI (0.807; 0.890) |  |  |  |  |  |
| 8. PSC | 0.564 CI (0.478; 0.638) | 0.718 CI (0.656; 0.774) | 0.863 CI (0.821; 0.901) | 0.737 CI (0.688; 0.782) | 0.922 CI (0.885; 0.955) | 0.789 CI (0.739; 0.834) | 0.811 CI (0.763; 0.856) |  |  |  |  |
| 9. FPQ | 0.588 CI (0.502; 0.663) | 0.405 CI (0.294; 0.508) | 0.527 CI (0.439; 0.606) | 0.371 CI (0.282; 0.454) | 0.479 CI (0.392; 0.557) | 0.498 CI (0.388; 0.594) | 0.517 CI (0.408; 0.610) | 0.510 CI (0.410; 0.598) |  |  |  |
