# Supplementary material for: Generative AI Engagement and Perceived Nutrition-Oriented Feeding Practices Among Urban Indonesian Mothers: Mixed Methods Study
Source: JMIR Pediatr Parent. 2026 Sep 21;9:e100061. doi: 10.2196/100061 (PMC13592562; doi:10.2196/100061)
Supplement: Supplementary file 5 [file pediatrics-v9-e100061-s006.docx]

**Multimedia Appendix 6**

**Table collinearity statistics (inner variance inflation factor values)**

| Predictor Constructs | Cognitive Empowerment (CE) | Parental Self-Compassion (PSC) | Perceived Feeding Practice Quality (FPQ) |
| --- | --- | --- | --- |
| AI Algorithmic Trust (AT) | 2.785 | 2.785 | - |
| AI Health Information Quality (IQ) | 1.376 | 1.376 | - |
| AI Personalization Fit Perception (PF) | 2.672 | 2.672 | - |
| AI Social Proof Sensitivity (SP) | 2.430 | 2.430 | - |
| AI Information Seeking Intensity (ISI) | 1.660 | 1.660 | - |
| Cognitive Empowerment (CE) | - | - | 2.673 |
| Parental Self-Compassion (PSC) | - | - | 2.231 |
| E-Health Self-Efficacy (ESE) | - | - | 2.549 |
| ESE × CE (Moderation) | - | - | 2.205 |
| ESE × PSC (Moderation) | - | - | 2.102 |
