# Supplementary material for: Generative AI Engagement and Perceived Nutrition-Oriented Feeding Practices Among Urban Indonesian Mothers: Mixed Methods Study
Source: JMIR Pediatr Parent. 2026 Sep 21;9:e100061. doi: 10.2196/100061 (PMC13592562; doi:10.2196/100061)
Supplement: Supplementary file 6 [file pediatrics-v9-e100061-s007.docx]

**Multimedia Appendix 7.** Bootstrapped specific and total indirect effects of AI-engagement constructs on perceived nutrition-oriented feeding practices.^a^

| **Pathway** | **Coefficient** | **95% CI** | ***t* value** | ***P* value** | **Result** |
| --- | --- | --- | --- | --- | --- |
| **Specific indirect effects** | | | | | |
| ISI → CE → NFP | 0.055 | 0.024 to 0.093 | 2.61 | 0.004 | **Indirect effect supported** |
| PF → CE → NFP | 0.064 | 0.027 to 0.116 | 2.43 | 0.007 | **Indirect effect supported** |
| SP → CE → NFP | 0.051 | 0.022 to 0.093 | 2.42 | 0.008 | **Indirect effect supported** |
| AT → PSC → NFP | 0.079 | 0.022 to 0.137 | 2.25 | 0.012 | **Indirect effect supported** |
| PF → PSC → NFP | 0.042 | 0.014 to 0.077 | 2.17 | 0.015 | **Indirect effect supported** |
| ISI → PSC → NFP | 0.026 | 0.009 to 0.050 | 2.13 | 0.017 | **Indirect effect supported** |
| IQ → CE → NFP | 0.042 | 0.014 to 0.085 | 1.98 | 0.024 | **Indirect effect supported** |
| IQ → PSC → NFP | 0.015 | 0.004 to 0.035 | 1.67 | 0.047 | **Indirect effect supported** |
| AT → CE → NFP^b^ | 0.023 | 0.005 to 0.058 | 1.49 | 0.068 | **Indirect effect supported** |
| SP → PSC → NFP^c^ | 0.002 | −0.008 to 0.016 | 0.25 | 0.402 | Indirect effect not supported |
| **Total indirect effects** | | | | | |
| PF → NFP | 0.105 | 0.064 to 0.161 | 3.64 | <.001 | **Indirect effect supported** |
| ISI → NFP | 0.081 | 0.048 to 0.123 | 3.62 | <.001 | **Indirect effect supported** |
| AT → NFP | 0.102 | 0.045 to 0.160 | 2.91 | 0.002 | **Indirect effect supported** |
| IQ → NFP | 0.058 | 0.029 to 0.097 | 2.73 | 0.003 | **Indirect effect supported** |
| SP → NFP | 0.052 | 0.022 to 0.094 | 2.43 | 0.008 | **Indirect effect supported** |

^a^Indirect effects were estimated by nonparametric bootstrapping (10,000 subsamples) in SmartPLS 4; confidence intervals are bias-corrected. A pathway is reported as supported when its bias-corrected 95% CI excludes zero. The structural model was specified as fully mediated on theoretical grounds; no direct AI-engagement-to-outcome paths were estimated, so mediation type is not classified and each supported pathway is reported as an indirect effect.

^b^The pathway AT → CE → NFP has a bias-corrected 95% CI that excludes zero although its two-tailed P value is .068; the bias-corrected interval was adopted as the primary inferential criterion for indirect effects.

^c^The pathway SP → PSC → NFP was not supported, consistent with the nonsupport of hypothesis H10.

Abbreviations: AT, AI Algorithmic Trust; IQ, AI Health Information Quality; PF, AI Personalization Fit Perception; ISI, AI Information Seeking Intensity; SP, AI Social Proof Sensitivity; CE, Cognitive Empowerment in Health Decision Making; PSC, Parental Self-Compassion in Feeding Context; NFP, Perceived Nutrition-Oriented Feeding Practices; CI, confidence interval.
